# Improving dietary quality in youth with type 1 diabetes: randomized clinical trial of a family-based behavioral intervention

**DOI:** 10.1186/s12966-015-0214-4

**Published:** 2015-05-08

**Authors:** Tonja R Nansel, Lori M B Laffel, Denise L Haynie, Sanjeev N Mehta, Leah M Lipsky, Lisa K Volkening, Deborah A Butler, Laurie A Higgins, Aiyi Liu

**Affiliations:** Health Behavior Branch, Division of Intramural Population Health Research, Eunice Kennedy Shriver National Institute of Child Health and Human Development, 6100 Executive Blvd. Rm 7B13R, MSC 7510 Bethesda, MD USA; Pediatric, Adolescent, and Young Adult Section, Genetics and Epidemiology Section, Joslin Diabetes Center, Harvard Medical School, Boston, MA USA

**Keywords:** Behavioral intervention, Nutrition, Diet, Type 1 diabetes, Children, Adolescents

## Abstract

**Background:**

Diets of children with type 1 diabetes are low in fruits, vegetables, and whole grains, and high in foods of minimal nutritional value, increasing risk for future adverse health outcomes. This 18-month randomized clinical trial tested the effect of a family-based behavioral intervention integrating motivational interviewing, active learning, and applied problem-solving on the primary outcomes of dietary intake and glycemic control among youth with type 1 diabetes.

**Methods:**

A parallel-group study with equal randomization was conducted at an outpatient, free-standing, multidisciplinary tertiary diabetes center in the United States. Eligible youth were those age 8–16 years with type 1 diabetes diagnosis ≥1 year and hemoglobin A1c (HbA1c) ≥6.5% and ≤10.0%. Participants were 136 parent-youth dyads (treatment n = 66, control n = 70). The intervention consisted of 9 in-clinic sessions delivered to the child and parent; control condition comprised equivalent assessments and number of contacts without dietary advice. Dietary intake was assessed using 3-day diet records at 6 time points across the 18-month study. Dietary outcomes included the Healthy Eating Index-2005 (HEI2005; index measuring conformance to the 2005 United States Dietary Guidelines for Americans) and Whole Plant Food Density (WPFD; number of cup or ounce equivalents per 1000 kcal of whole grains, whole fruit, vegetables, legumes, nuts, and seeds consumed). HbA1c was obtained every 3 months. Overall comparison of outcome variables between intervention and usual care groups was conducted using permutation tests.

**Results:**

There was a positive intervention effect across the study duration for HEI2005 (p = .015) and WPFD (p = .004). At 18 months, HEI2005 was 7.2 greater (mean ± SE 64.6 ± 2.0 versus 57.4 ± 1.6), and WPFD was 0.5 greater (2.2 ± 0.1 versus 1.7 ± 0.1) in the intervention group versus control. There was no difference between groups in HbA1c across the study duration.

**Conclusions:**

This behavioral nutrition intervention improved dietary quality among youth with type 1 diabetes, but did not impact glycemic control. Findings indicate the potential utility of incorporating such strategies into clinical care, and suggest that improvement in diet quality can be achieved in families living with this burdensome disease.

**Trial registration:**

Clinicaltrials.gov registration number: NCT00999375

**Electronic supplementary material:**

The online version of this article (doi:10.1186/s12966-015-0214-4) contains supplementary material, which is available to authorized users.

## Introduction

Medical nutrition therapy is an integral component of diabetes management education to facilitate optimal glycemic control and prevention of complications [1]. Nutrition education for this population includes education on carbohydrate estimation as well as recommendations for general healthful eating [1,2]. Nevertheless, the diets of youth with type 1 diabetes (T1D) are characterized by patterns known to increase risk for certain chronic diseases [3]. Intake of fruits, vegetables, and whole grains are far below dietary recommendations [4,5]. Intake of total and saturated fat is above recommendations [6,7], and a substantial proportion of daily energy intake is obtained from refined grains and discretionary foods such as chips and sweets [5].

Previous research indicates the critical role of diet in promoting long-term health among persons with type 1 diabetes, including reducing risk of cardiovascular disease. Cardiovascular disease is more common, occurs earlier, and is the primary cause of premature mortality in persons with T1D [8,9]. This increased risk begins early in the disease process, with children and adolescents with T1D demonstrating subclinical cardiovascular abnormalities [9]. In observational studies among persons with T1D, better diet quality is associated with lower blood pressure [10], more optimal LDL/HDL ratio [11], and lower CVD risk profile including lower arterial stiffness [12]. In a study of youth with T1D in Italy, implementation of a Mediterranean-style diet led to improved lipid profiles [13]. Considering the high prevalence of cardiovascular risk factors observed in youth with T1D [14-18], optimal dietary intake is critical for improving long-term health outcomes among this population. The effect of diet quality on glycemic control, however, is not well-established. In an observational study of youth with T1D, better diet quality was associated with lower A1c [19], and in short-term feeding studies, better diet quality has been shown to improve glycemic control [20,21].

Despite suboptimal diet quality among youth with type 1 diabetes and the potential long-term health benefits of improving dietary intake, little previous research has addressed strategies for improving dietary intake in this population. Limited research has tested the efficacy of specific dietary recommendations on health outcomes, including evaluating the effect of a Mediterranean-style diet on lipid profile [13], the effect a low glycemic index diet on glycemic control [22], and the effect of an optimized mixed diet on dietary nutrient composition [23]. These studies utilized educational guidance only; however, it is well-established that optimal methods for achieving dietary change incorporate behavioral strategies along with educational guidance [24]. Behavioral strategies such as self-monitoring, goal-setting, problem-solving, contracting, and motivational interviewing have demonstrated effectiveness in achieving healthful dietary change in youth in the general population [24]. Additionally, achieving dietary change among youth must consider the key role played by parents, who influence youth’s dietary behavior through behaviors such as modeling eating habits and determining what foods are available and accessible in the home [25]. To date, no randomized trial of a behavioral intervention to improve dietary intake among youth with type 1 diabetes has been published. The purpose of this study was to evaluate the efficacy of a family-based behavioral intervention that integrated motivational interviewing, active learning, and applied problem-solving to increase intake of whole plant foods (fruit, vegetables, whole grains, legumes, nuts and seeds) among youth with type 1 diabetes. We hypothesized that the intervention would improve youth diet quality and glycemic control relative to the control condition.

### Subjects and methods

#### Design and participants

This was a parallel-group study with equal randomization conducted at an outpatient, free-standing, multidisciplinary tertiary diabetes center in Boston, Massachusetts. Eligibility criteria included age 8.0 to 16.9 years, diagnosis of type 1 diabetes ≥ 1 year, daily insulin dose ≥0.5 units per kilogram, most recent HbA1c ≥6.5% and ≤10.0%, intensive insulin therapy with either an insulin regimen of ≥3 injections daily or insulin pump, at least one clinic visit in the past year, and ability to communicate in English. Exclusion criteria included daily use of premixed insulin, transition to insulin pump therapy in the last three months, real-time continuous glucose monitoring use in the last three months, participation in another intervention study in the last six months, and presence of gastrointestinal disease such as celiac disease, multiple food allergies, use of medications that interfere significantly with glucose metabolism, or significant mental illness.

#### Procedures

The study was conducted from August 2010 through May 2013. Medical record data were screened to identify eligible patients; recruitment was implemented by trained research staff at regular clinic visits. All youth provided assent; parents and youth turning 18 years old during the 18-month trial provided written informed consent. Randomization was stratified by age (<13 years and ≥13 years), HbA1c (<8.5% and ≥8.5%), and insulin regimen (injection and insulin pump), with a permuted block randomization scheme. Randomization was conducted by the data coordinating center; group assignment was indicated to the site research assistant by an online data management system, and families were informed of their group assignment at the second study visit.

Families were enrolled in the study for 18 months. Study visits were completed in the clinic; diet records were completed in the home following assessment visits. Youth and parents each received a total of $380 compensation for completion of all study visits and reimbursement for parking costs. Study procedures followed were approved by the *Eunice Kennedy Shriver* National Institute of Child Health and Human Development Institutional Review Board and the Joslin Diabetes Center Committee on Human Subjects.

#### Treatment conditions

The intervention content and process was guided by self-regulation perspective [26], social cognitive theory [27], and self-determination theory [28]. Each session integrated a motivational interviewing style of interaction designed to increase internal motivation for healthful eating [29,30], active learning for youth and parents to facilitate skill-building and engagement with the educational information, and applied problem-solving to facilitate goal-directed behavior and self-regulation skills. The intervention was delivered by research assistants who received training in pediatric T1D, intervention procedures, and motivational interviewing. In addition, study investigators provided feedback on audiotaped role-play practice sessions prior to intervention delivery and on a random sample of audiotaped intervention sessions.

Families in the intervention condition received six “core” sessions during the first seven months of the study period. An initial overview session addressed key principles of healthy eating, with a focus on increasing intake of whole plant foods, defined as whole fruits, vegetables, whole grains, legumes, nuts, and seeds. These food groups were emphasized due to their importance in the diet for disease prevention and consistent findings of low intake relative to dietary guidelines [31-33]. Monitoring of carbohydrate intake is central in T1D management [1,2]; the focus on these food groups encourages families to also consider the quality of their sources of carbohydrate. The next five sessions addressed application of these principles to specific eating contexts – breakfast, lunch, dinner, snacks, and eating out (one context per session). Each session included interactive education, learning activities, goal-setting, and application of the problem-solving process to increase intake of fruits, vegetables, whole grains, and/or legumes/nuts/seeds at that eating occasion. Children and parents set goals for increasing intake of two selected food groups, considered barriers, chose strategies, and developed a specific action plan for increasing their intake of the target foods. At each session, families reviewed their progress on the previous session’s goal, allowing previous efforts to inform subsequent problem-solving. Three “booster” sessions delivered during months nine to fifteen dealt with overcoming challenges associated with social eating, meal planning, and the food environment. Families were provided with a book of approximately 300 recipes highlighting the target food groups and providing detailed nutrition information to assist with insulin dosing. Recipes were selected based on consideration for ease of preparation, acceptability and familiarity. Intervention materials are summarized in the Additional file 1: Table S1 and are available upon request from the corresponding author.

Participants in both groups received intermittent, masked continuous glucose monitoring (CGM) for three consecutive days six times across the study duration, paired with completion of diet records. Following completion of each monitoring period, all subjects received individualized feedback on their CGM results with a diabetes nurse educator or certified diabetes educator. For intervention families, CGM feedback reinforced session content by addressing how glycemic patterns were associated with quality of food ingested, highlighting the effect of food choices on blood glucose levels.

The control condition was designed to match on potentially important aspects of research contact that may impact health outcomes but were not the focus of the behavioral intervention. Participants in the control condition received equal frequency of contacts with research staff, focused on case management (scheduling, confirming, and documenting medical follow-up) within the diabetes health care system in a “care ambassador” model [34], and equal frequency of three-day masked CGM use. Participants in the control condition received no additional dietary advice beyond that provided as part of standard type 1 diabetes care. Scales, measuring cups, and spoons were also provided to all participants to facilitate portion size estimation.

#### Measures

The child’s usual dietary intake was estimated using three-day food records. Children and parents were instructed on accurately measuring and reporting food and beverage intake and given a sample diet record. Families were instructed to keep records beginning at the time of CGM insertion and continuing for the next three consecutive days. Families were asked to use measuring utensils when at home, and if away from home, to provide their best estimate of portion size. Families were reminded to provide all specific details for each food item, including names of brands or restaurants and specific item labeling (e.g., low fat, 1% milk), and to leave no blank fields on the form. Research staff reviewed the completed records upon receipt from the family to ensure completeness, and solicited missing information (e.g., brand names) from the family as needed. For visits in which a family did not complete a diet record, 2 non-consecutive 24-hour dietary recalls were obtained by a registered dietician (1.7% of dietary assessments). Diet records were entered by two registered dietitians and verified for consistency and accuracy. Nutrition Data System for Research software (NDSR 2012; Nutrition Coordinating Center, University of Minnesota, Minneapolis, MN) was used to analyze the records and assess nutrient intake and food group servings.

Hemoglobin A1c (HbA1c) was measured using a laboratory assay standardized to the Diabetes Control and Complications Trial (reference range, 4%-6%, [20–42 mmol/mol]). Initial A1c assays were performed with the Tosoh (Tosoh Medics, South San Francisco, CA, USA) followed by the Roche Cobas Integra (Indianapolis, IN). All values obtained with the Tosoh were standardized to the Roche assay. Height, weight, insulin regimen, and frequency of blood glucose monitoring were extracted from the medical records. Demographic characteristics were assessed by parent self-report. The poverty income ratio was calculated as the ratio of reported household income divided by the 2008 US Census poverty threshold for household size and composition adjusted for inflation [35]. This measure accounts for household size when evaluating income, with a higher value indicating greater income.

#### Primary outcomes and power

Primary study outcomes were diet quality and glycemic control. Two indicators of overall diet quality were evaluated. The Healthy Eating Index 2005 (HEI2005) score measures conformance to the 2005 Dietary Guidelines for Americans, and is comprised of 12 component scores corresponding to dietary guidelines for intake of total fruit, whole fruit, total vegetables, dark green/orange vegetables and legumes, total grains, whole grains, milk, meat and beans, oils, saturated fat, sodium and energy from solid fat, alcohol and added sugars [36]. The maximum component score is achieved if intake meets recommended intake levels, with truncation for intakes exceeding recommendations. Recommendations and scores are expressed on a per-1000 kilocalorie basis to enable comparability and applicability to individuals regardless of total energy requirements. Component scores are summed to obtain the total score, with possible values ranging from 0–100; a score of 100 indicates meeting intake recommendations for all dietary components. Whole Plant Food Density (WPFD) is a continuous measure that represents the proportion of the diet allocated to whole grains, whole fruit, vegetables, legumes, nuts, and seeds; calculated as the total number of cup or ounce equivalents of these foods consumed per 1000 kilocalorie total intake [37]. WPFD was developed by two of the investigators to provide a measure that directly corresponds to the target food groups of the intervention.

A target sample size of 160 participants was selected based on detecting meaningful differences between intervention and control conditions in dietary intake and HbA1c at 18-month follow-up. At the time of the study development, there were no published data quantifying HEI2005 scores in a cohort of youth with T1D, and the WPFD had not yet been developed. Based on available data from 67 youth age 2 to 12 years receiving care at the same source population (mean ± SD HEI2005 57.6 ± 6.5), the sample size provided 97% power to detect a 4 point difference in the HEI2005. Power for detecting treatment effect on HbA1c was based on electronic medical record data from 560 patients at the recruiting clinic site, ages 8–16 years, with HbA1c between 7.5 and 9.5%, inclusive (mean ± SD HbA1c 8.4 ± 0.6); the target sample provided 88% power to detect a 0.3% difference in HbA1c. Given the small sample size used for power analyses of dietary outcomes, and the later development of the WPFD, power analyses were subsequently recalculated using data from a larger cross-sectional study of subjects from the same source population [5]. Using a two-sample *t*-test with a two-sided 5% significance level, the power at the achieved sample size of 136 was 83% to detect a difference between groups of 5.5 in HEI2005, 86% to detect a difference between groups of 0.7 in WPFD, and 93% to detect a difference between groups of 0.5% in HbA1c, assuming a common standard deviation across groups of 10.95 for HEI2005, 1.33 for WPFD, and 0.84 for HbA1c.

#### Analysis

Baseline demographic and disease-related characteristics of the study participants were summarized with means and standard deviations for continuous variables and frequencies for categorical/ordinal variables. Comparison of these variables between intervention and control groups was done using independent t-tests for continuous variables or Pearson chi-square for categorical variables.

Mean values for each dietary outcome variable (overall diet quality indicators and individual food groups) at each visit for each treatment condition were estimated by population ratios, which take the ratio of total food group intake to total energy intake at the population (treatment group) level; this method reduces bias in estimates of usual intakes from limited dietary assessment data [38]. The standard errors of each study outcome were estimated using bootstrap with 5000 samples with replacement. Between-group comparison of each outcome was conducted using permutation test. Five-thousand permutations of the combined samples were generated to obtain the permuted p-value, that is, the proportion of the permuted samples that yielded a more extreme difference than that observed between intervention and control conditions. Here the difference was defined as the Euclidean distance in the vector of visit-specific population ratios between intervention and control groups; the resulting p-value indicates significance of between-group differences across the study duration. Intent-to-treat analyses were conducted using multiple imputation for missing data, including missing due to subject withdrawal. Analyses were applied to ten complete data sets obtained by replacing the missing outcome values with imputation. Estimates from the imputed samples were then combined to generate a single estimate and p-value [39]. A p-value of less than 0.05 was considered statistically significant. All analyses were performed using either SAS version 9.3 (SAS Institute, Cary, NC) or R version 2.15.1 (The R Foundation for Statistical Computing).

## Results

**Figure 1:**
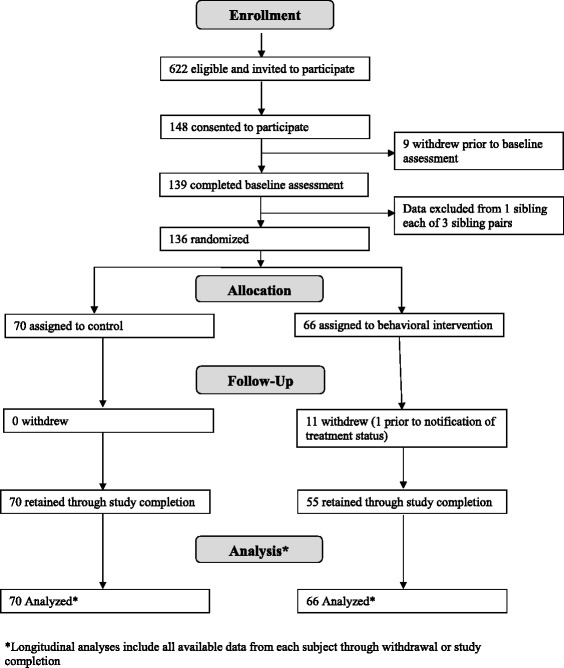
Participant flow through a randomized clinical trial of a family-based behavioral intervention to improve diet quality in youth with type 1 diabetes.

Participant flow from recruitment through follow-up is reported in Figure 1. Of those invited, 24% provided informed consent and 22% completed baseline. Subject retention through study completion was 92%. All subjects who withdrew had been randomized to the intervention group. One subject withdrew after baseline but before being informed of treatment assignment, 2 withdrew within the first 3 study months; 3 between months 3 and 6, 1 between months 6 and 9, 3 between months 9 and 12, and 1 after month 12. Reasons for withdrawal were primarily lack of time to participate. No study-related adverse events were reported.

Baseline characteristics were well-balanced between groups (Table 1). Approximately two-thirds of the sample used insulin pump therapy. The mean HbA1c was 8.1%. Mean intake of fruit, vegetables, and whole grains was well below dietary guidelines.

**Table 1:** **Sample characteristics of youth with type 1 diabetes participating in a behavioral nutrition intervention trial (N = 136)**

	**All participants**	**Treatment (N = 66)**	**Control (N = 70)**	**p** ^**1**^
**Demographics**	**Mean ± SD or N (%)**	**Mean ± SD or N (%)**	**Mean ± SD or N (%)**	
Age (years)	12.8 ± 2.6	12.6 ± 2.7	13.0 ± 2.5	0.27
Sex				
Male	66 (48.5)	35 (53.0)	31 (44.3)	0.31
Female	70 (51.5)	31 (47.0)	39 (55.7)	
Race/ethnicity				
White, non-Hispanic	123 (90.4)	58 (87.9)	65 (92.9)	0.17
Hispanic	7 (5.2)	6 (9.1)	1 (1.4)	
Black	5 (3.7)	2 (3.0)	3 (4.3)	
Other	1 (0.7)	0 (0.0)	1 (1.4)	
Highest parent education level^2^				
High school or equivalent	8 (5.9)	4 (6.1)	4 (5.7)	0.48
Junior college, technical or some college	27 (19.9)	11 (16.7)	16 (22.9)	
College degree	46 (33.8)	20 (30.3)	26 (37.1)	
Graduate education	55 (40.4)	31 (47.0)	24 (34.3)	
Family poverty income ratio^2^	5.2 ± 3.1	5.5 ± 3.2	4.9 ± 3.0	0.23
**Diabetes and health-related characteristics**				
Duration of diabetes (years)	6.0 ± 3.1	5.6 ± 2.5	6.3 ± 3.6	0.15
Insulin regimen				
Injection only	42 (30.9)	20 (30.3)	22 (31.4)	0.89
Pump	94 (69.1)	46 (69.7)	48 (68.6)	
Frequency of blood glucose monitoring (times/d)	5.7 ± 2.4	5.8 ± 2.4	5.6 ± 2.5	0.60
Hemoglobin A1c (%)	8.1 ± 1.0	8.1 ± 1.1	8.1 ± 1.0	0.95
BMI z-score	0.68 ± 0.82	0.65 ± 0.81	0.71 ± 0.84	0.65
**Dietary Intake**				
% kcal from carbohydrate	47.9 ± 0.5	48.0 ± 0.7	47.9 ± 0.7	0.93
% kcal from protein	16.2 ± 0.2	16.1 ± 0.4	16.3 ± 0.4	0.70
% kcal from fat	35.9 ± 0.5	35.9 ± 0.7	35.8 ± 0.6	0.92
Whole plant food density	1.89 ± 0.09	1.87 ± 0.13	1.91 ± 0.13	0.84
Fruit (cup equivalents per 1000 kcal)	0.28 ± 0.03	0.29 ± 0.04	0.26 ± 0.03	0.60
Vegetable (cup equivalents per 1000 kcal)	0.53 ± 0.03	0.50 ± 0.04	0.55 ± 0.04	0.38
Whole grains (ounce equivalents per 1000 kcal)	0.69 ± 0.05	0.66 ± 0.07	0.72 ± 0.08	0.59
Legumes, nuts, and seeds (cup equivalents per 1000 kcal)	0.17 ± 0.03	0.17 ± 0.04	0.17 ± 0.03	1.00
Healthy eating index 2005	57.3 ± 1.3	57.3 ± 1.8	57.2 ± 2.0	0.99

^1^Comparisons between intervention and control groups using independent *t*-tests for continuous variables or chi-square for categorical variables.

^2^Missing data from 1 participant on highest parent education and from 2 participants on family income.

**Figure 2:**
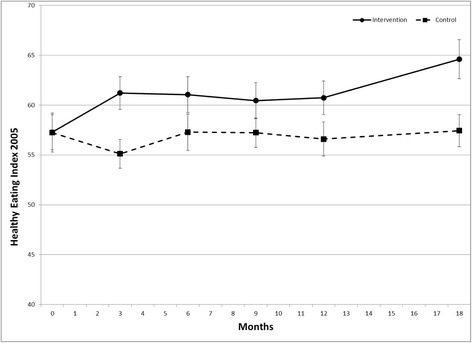
Effect of a dietary behavioral intervention on Healthy Eating Index 2005 in youth with type 1 diabetes.

**Figure 3:**
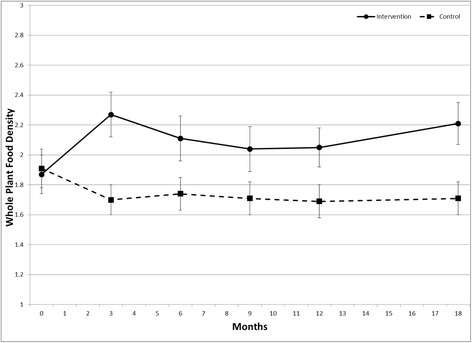
Effect of a dietary behavioral intervention on Whole Plant Food Density in youth with type 1 diabetes.

**Figure 4:**
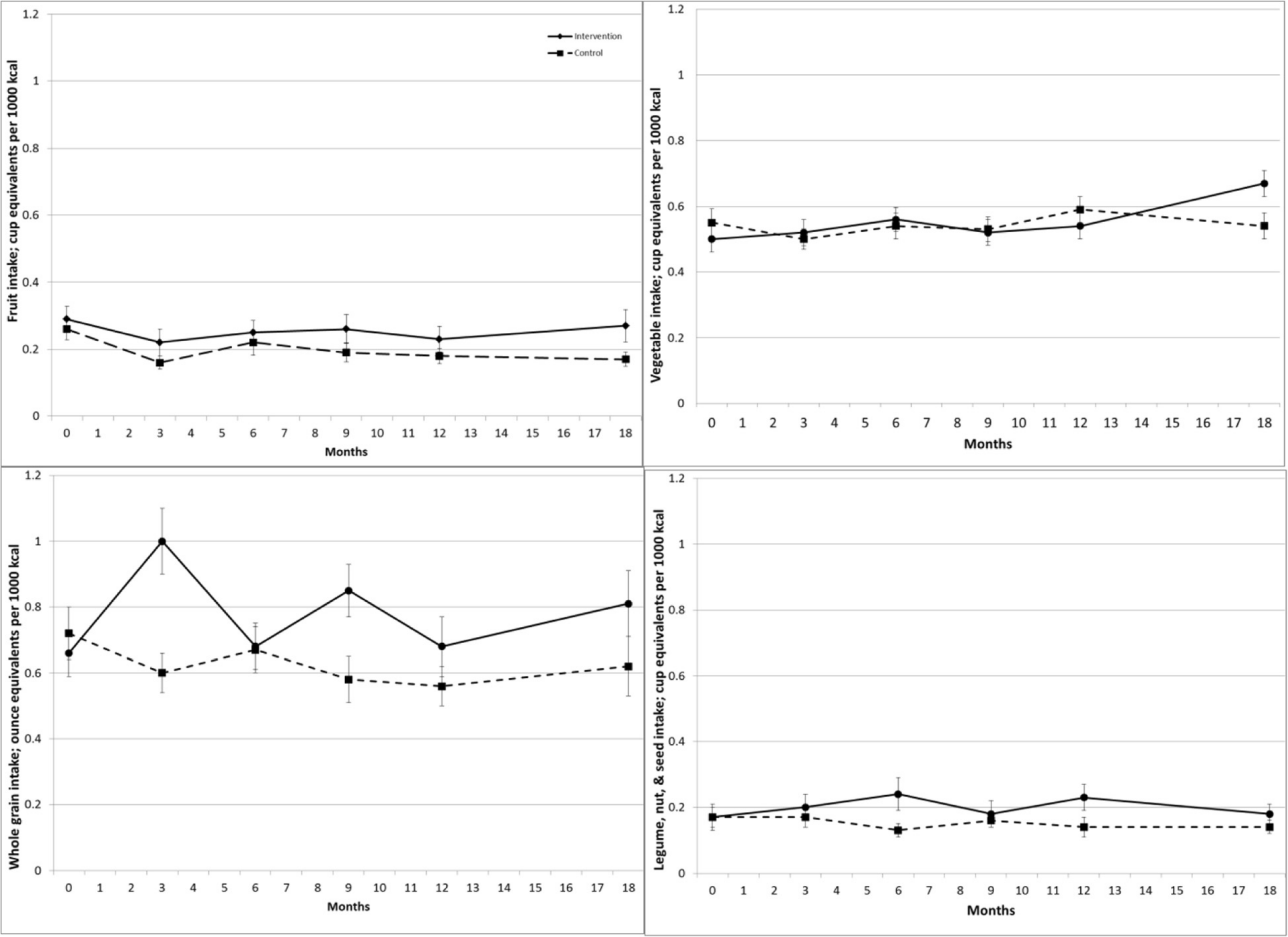
Effect of a dietary behavioral intervention on intake of whole fruit, vegetables, whole grains, and legumes/nuts/seeds in youth with type 1 diabetes.

**Figure 5:**
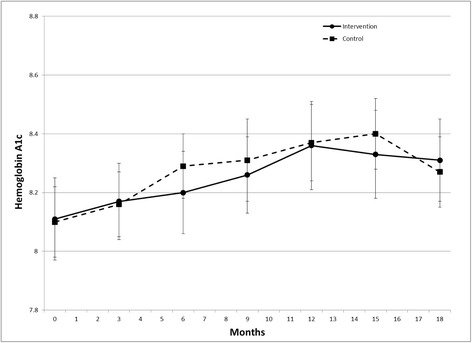
Effect of a dietary behavioral intervention on glycemic control in youth with type 1 diabetes.

Intervention effects on diet quality are shown in Figures 2 and 3. There was a positive intervention effect across the study duration (permutation test) for HEI2005 (p = .015) and WPFD (p = .004). At 18 months, HEI2005 was 7.2 greater (mean ± SE 64.6 ± 2.0 versus 57.4 ± 1.6), and WPFD was 0.5 greater (2.2 ± 0.1 versus 1.7 ± 0.1) in the intervention group versus control. Analysis by individual target food groups (Figure 4) indicated greater intake of whole grains among the treatment group (p = .003), but no difference in the intake of the other individual food groups. Treatment groups did not differ in the percent of energy intake obtained from carbohydrate or fat across the study duration (data not shown). There was no difference between groups in HbA1c across the study duration (Figure 5).

## Discussion

This dietary behavioral intervention demonstrated efficacy in improving overall diet quality and increasing intake of whole plant foods among youth with type 1 diabetes. At 18-month follow-up, youth in the intervention group had a mean HEI2005 score more than 7 points higher and consumed half a cup or ounce equivalent per 1000 kilocalorie more whole plant foods than youth in the control group. The difference in HEI2005 attributable to the intervention is comparable to a 1-quintile difference in HEI2005 as observed in the Nurses’ Health and Health Professionals Follow Studies, in which HEI2005 was linearly associated with chronic disease risk [40]. Only one previous intervention reported impact on HEI2005 scores; this study was conducted in adults, and observed increases in up to approximately 3 points in treatment and control groups, but no discernible intervention effect [41]. To our knowledge, this is the first study to evaluate the impact of a dietary intervention on HEI2005 in a pediatric population. Notably, HEI2005 and WPFD demonstrated improvement from months 12–18, during which time the intensity of the intervention had decreased. This finding could reflect to a cumulative effect of repeated engagement in the problem-solving process, or the timing and content of the booster sessions, which focused on integrating dietary changes into the families’ lifestyle.

Examination of the food groups targeted by the intervention indicated a significant effect on increasing whole grain intake. A growing body of research documents the benefit of whole grain intake on cardiovascular and other chronic disease risk indicators [42,43]. Few previous dietary interventions for youth have addressed whole grain intake, and there is little data on the acceptability of whole grains among youth. The observed intervention effect on whole grain intake was greater than that observed in two recently-reported interventions in youth [44,45]. In another study, [46] a larger intervention effect on whole grain intake (approximately 3 ounce equivalents) was observed; however, the study duration was only 6 weeks, and maintenance of this effect is unknown. Importantly, in contrast to the present study, these previous interventions provided participants with whole grain foods to consume, and no assessment occurred once the foods were no longer provided. In the current study, intervention materials were designed to highlight a variety of whole grain products and assist families in identifying whole grain products through label reading and interpretation of the whole grain stamp. Families were encouraged to try a variety of whole grains to find those that they enjoyed. Findings suggest that whole grain products can be acceptable to youth and indicate the feasibility of increasing their intake.

The difference between groups in fruit intake across the study duration was not statistically significant. Two recent systematic reviews and meta-analyses reported a mean increase of approximately 0.5 daily servings of fruit and vegetables in children participating in behavioral and school-based interventions [47,48], suggesting that further research is needed to determine ways to increase intake of these foods in youth with T1D. Previous research has demonstrated lower intake of fruit among youth with T1D than among the general population [4] and families may limit fruit intake due to erroneous concerns about the effect of fruit on blood sugar levels [49]. Although the intervention included information to correct such possible concerns and facilitate accurate estimation of carbohydrate content for fruits, these issues may represent barriers to increasing fruit intake nonetheless. The intervention did not affect vegetable intake. Previous dietary interventions in youth in the general population have similarly shown little or no effect on vegetables intake [50]. Findings suggest a need for further development of effective methods to promote vegetable intake among youth.

Previous research suggests that improving diet quality may facilitate greater glycemic control [20,21]. However, this dietary behavioral intervention did not have a positive or negative effect on glycemic control. It is possible that the degree of difference in dietary intake between groups observed in this study may not have been sufficient to directly impact glycemic control. Additionally, families in both groups received intermittent masked CGM with feedback, which could have modestly impacted glycemic control. Finally, this negative finding with respect to glycemic control may reflect the substantial difficulties related to improving HbA1c in pediatric patients with T1D. Nevertheless, improving diet quality in this population is of importance regardless of impact on glycemic control, given the role of healthful dietary intake in reducing risk for adverse cardiovascular outcomes [10-13] and other complications [51].

Findings should be interpreted in light of the study limitations. The sample was drawn from a single clinic with a limited number of minority and low-income families, and resulted in a 24% recruitment rate, both of which limit the generalizability of these results to the general population of youth with T1D. The mean HEI2005 at baseline in this sample was 57.3, which is slightly higher than that reported in the US general population of youth (54.7 for those aged 6 to 11 years and 54.8 for those aged 12 to 17 years) [31]. Considering the observed recruitment rate, this difference could reflect possible sampling bias, as families consuming a healthier diet may have had greater interest in study participation. Families who elected to participate were well-retained throughout the study duration; however, all participant withdrawals were in the intervention group. This may be attributable to the added time burden of participation in the intervention sessions. Intent-to-treat analysis was used to minimize potential bias due to unequal withdrawal across groups. While diet records are among the most reliable and valid measures of dietary intake, it is a burdensome method, and the task of completing food records may influence intake. However, food records capture diet with great detail relative to food frequency questionnaires or diet screeners, and are less susceptible to recall bias [52]. Parents and children were trained together in the completion of the diet records to address the developmental and practical needs of this population.

## Conclusions

Despite the documented poor diet quality among youth with T1D and the importance of dietary intake in disease management and prevention of long-term complications, particularly cardiovascular disease, little research has addressed behavioral strategies for improving dietary intake in this high-risk population. This study demonstrated the efficacy of a theoretically-grounded, family based behavioral intervention integrating motivational interviewing, active learning, and applied problem-solving for improving diet quality among youth with T1D. Findings indicate the potential utility of incorporating such strategies into clinical care, and suggest that improvement in diet quality can be achieved in families living with burdensome disease.

## Additional file

Additional file 1: Table S1. Intervention Summary.

## Abbreviations

HbA1c: Hemoglobin A1c
CGM: Continuous glucose monitoring
HEI2005: Healthy Eating Index 2005
T1D: Type 1 diabetes
WPFD: Whole Plant Food Density
