# Supplementary material for: Improving dietary quality in youth with type 1 diabetes: randomized clinical trial of a family-based behavioral intervention
Source: Int J Behav Nutr Phys Act. 2015 May 8;12:58. doi: 10.1186/s12966-015-0214-4 (PMC4436744; doi:10.1186/s12966-015-0214-4)
Supplement: Additional file 1: Table S1. — Intervention Summary. [file 12966_2015_214_MOESM1_ESM.docx]

**Table S1: Intervention Summary**

| **Healthy Eating - Eat Well, Live Well**   - Interactive Education – target food groups as “food superstars”; typical intake versus dietary recommendations - Activity – identification of individual’s top reasons for eating healthy - Activity – readiness to increase intake of target food groups - Interactive Education - fruits and vegetables serving sizes - Activity – try something new - Activity – selection of a concrete, simple first healthy eating goal |
| --- |
| **Breakfast**   - Interactive Education – review of reasons for eating healthy; review of previous goal and progress - Activity – breakfast around the world - Interactive Education – breakfast basics - Interactive Education – navigating the grocery aisle: cereals - Activity – evaluation of typical breakfasts relative to target food groups; selection of improved breakfast options - Interactive Education – build up your breakfast (breakfast substitutions) - Goal-setting/problem-solving process for target foods at breakfast (goals, barriers, strategies, action plan, rewards) |
| **Lunch**   - Interactive Education – review of previous goal and progress - Interactive Education – lunch basics - Interactive Education – facts about fats - Interactive Education – navigating the grocery aisle: breads - Activity – kitchen stadium challenge; developing lunch recipe using target food groups - Activity – evaluation of typical lunches relative to target food groups; selection of improved lunch options - Interactive Education – upgrade your lunch (lunch substitutions) - Interactive Education – well stocked pantry - Goal-setting/problem-solving process for target foods at lunch (goals, barriers, strategies, action plan, rewards) |
| **Dinner**   - Interactive Education – review of previous goal and progress - Interactive Education – dinner basics - Interactive Education – navigating the grocery aisle: finding whole foods - Interactive Education – don’t let it go to waste (preventing spoilage) - Interactive Education – the plate method - Interactive Education – vegetables - Activity – evaluation of typical dinners relative to target food groups; selection of improved dinner options - Interactive Education – dinner makeovers (dinner substitutions) - Goal-setting/problem-solving process for target foods at dinner (goals, barriers, strategies, action plan, rewards) |
| **Snacks**   - Interactive Education – review of previous goal and progress - Activity – snack attack - Interactive Education – snack basics - Interactive Education – nuts about nuts - Interactive Education – navigating the grocery aisle: snack bars - Activity – evaluation of typical snacks relative to target food groups; selection of improved snack options - Interactive Education – healthy snack substitutions - Goal-setting/problem-solving process for target foods at snacks (goals, barriers, strategies, action plan, rewards) |
| **Restaurants**   - Interactive Education – review of previous goal and progress - Activity – restaurant trivia - Interactive Education – restaurant challenges & solutions; navigating the restaurant menu - Interactive Education – best restaurant choices (restaurant substitutions); healthy world cuisines - Activity – CHEF family restaurant; selecting healthful menu options highlighting target food groups - Goal-setting/problem-solving process for target foods at restaurants (goals, barriers, strategies, action plan, rewards) |
| **Social Eating**   - Interactive Education – review of previous goal and progress - Interactive Education – social eating discussion questions - Interactive Education – social eating challenges - Interactive Education – social eating strategies - Goal-setting/problem-solving process for target foods in social situations (goals, barriers, strategies, action plan, rewards) |
| **Meal Planning**   - Interactive Education – review of previous goal and progress - Interactive Education – meal planning discussion questions - Interactive Education – meal planning challenges - Interactive Education – meal planning strategies - Goal-setting/problem-solving process for target foods related to meal planning (goals, barriers, strategies, action plan, rewards) |
| **Food Environment**   - Interactive Education – review of previous goal and progress - Interactive Education – food environment discussion questions - Interactive Education – food environment challenges - Interactive Education – food environment strategies - Goal-setting/problem-solving process for target foods related to food environment (goals, barriers, strategies, action plan, rewards) |
